# Supplementary material for: Knowledge and coping style about depression in medical students: A cross-sectional study in China
Source: PLoS One. 2023 Oct 27;18(10):e0293511. doi: 10.1371/journal.pone.0293511 (PMC10610079; doi:10.1371/journal.pone.0293511)
Supplement: S2 File — (DOCX) [file pone.0293511.s002.docx]

Questionnaire on depression in medical college students

Dear:

We are the research team of Changzhi Medical College. We developed the questionnaire to understand medical college students' cognition of depression and the degree of depression.

This questionnaire adopts the survey method of anonymous participation. Please read each question carefully and answer it truthfully according to your actual situation.

Are you still willing to participate in our survey after read and understand our research intentions?

□yes □no

Please read each question carefully and answer it truthfully according to your actual situation.

1. Please choose Your Specialty

□Clinical medicine

□Nursing

□Oral medicine

□Anesthesiology

□Preventive medicine

□Pharmacy

□Medical imaging

□Rehabilitation Therapeutics

□Medical technology

□Biomedical engineering

□Information management and system

1. Please choose your grade

□Freshman □Sophomore □ Junior □ Senior □internship

1. Please choose your age

□18 □19 □ 20 □ 21 □22 □23 □24 □25

1. Please choose your gender

□male □female

1. Please choose your place of residence

□Urban □Rural-urban □Rural

1. Do you come from One-child family?

□yes □no

1. Do you think “you have good family relationship”?

□yes □no

1. Do you think“depression is a disease ”?

□yes □no

1. Do you think “depression can be cured by oneself”?

□cannot

□mild level depression can be cured

□mild and moderate level depression can be cured

□all level depressioncan be cured

10.Do you think “depression associated with genetic factor”?

□yes □no

1. Do you think “female have more probability suffered depression”?

□yes □no

1. Do you think “depression associated with [hormone](C:/Users/Administrator/AppData/Local/Yodao/DeskDict/frame/20201115211710/javascript:void(0);) [secretion](C:/Users/Administrator/AppData/Local/Yodao/DeskDict/frame/20201115211710/javascript:void(0);)?”

□yes □no

1. Do you think “depression associated with chronic disease?”

□yes □no

1. Do you think “depression associated with one`s personality?

□yes □no

1. Do you know suicide rate with depression in China?

□know □do not know □not clear

1. Do you know about symptom of depression?

□yes □no □no clear

1. Do you think “depression associated with drug abuse?”

□yes □no

1. Do you know about treatment method of depression?

□yes □no □no clear

1. Do you know about drug use of depression?

□yes □no □no clear

1. Do you think “depression can relapse”?

□can □cannot

1. Do you know about side-effect of depression cure?

□yes □no □no clear

1. Do you know about depression [influence](C:/Users/Administrator/AppData/Local/Yodao/DeskDict/frame/20201115211710/javascript:void(0);) for health?

□yes □no □no clear

1. Do you know incidence of depression in college students?

□know □do not know

1. Do you think “depression can be adjusted by oneself”?

□yes □no

1. Do you know about prevention method of depression?

□know □do not know □not clear

1. Zung Self-Rating Depression Scale

Intructions: for each item below, please place a check mark (√) in the column which best describes how often you felt or behaved this way during the past several days.

|  | a little of the time | some of the time | a large part of the time | most of the time |
| --- | --- | --- | --- | --- |
| I feel down-hearted and blue |  |  |  |  |
| I am restless and cannot keep calm |  |  |  |  |
| I have crying spells or feel like it |  |  |  |  |
| I have trouble sleeping at night |  |  |  |  |
| I am more irritable than usual |  |  |  |  |
| I feel that others would be better if I were dead |  |  |  |  |
| I noticed that I am losing weight |  |  |  |  |
| I have trouble with constipation |  |  |  |  |
| My heart beats faster than usual |  |  |  |  |
| I feel tired for no reason |  |  |  |  |
| My mind is as clear as usual |  |  |  |  |
| I still enjoy the things I used to do |  |  |  |  |
| Morning is when the best in all day |  |  |  |  |
| I feel hopeful about the future |  |  |  |  |
| I eat as much as usual |  |  |  |  |
| I find it easy to make decisions |  |  |  |  |
| I feel that I am usual and needed |  |  |  |  |
| My life is pretty full |  |  |  |  |
| I still enjoy happy when contact with others |  |  |  |  |
| I find it easy to do the things I used to |  |  |  |  |

1. If you suspect you have depression, how will you solve it?(multiple choice)

□No coping way

□Ask for help from friends

□Search a book

□Ask for help from psychological consultation office in the college

□See the doctor to hospital

Thank you for your work!
