# Supplementary material for: Knowledge and coping style about depression in medical students: A cross-sectional study in China
Source: PLoS One. 2023 Oct 27;18(10):e0293511. doi: 10.1371/journal.pone.0293511 (PMC10610079; doi:10.1371/journal.pone.0293511)
Supplement: Attachment — Submitted filename: Response to Reviewers.docx [file pone.0293511.s004.docx]

**Our responds to the** **editor’s and the reviewer’s comments：**

**Reply to editor**

We thank you very much for the constructive comments and suggestions on our manuscript. We have corrected our manuscript according to the comments.

1. Please ensure that your manuscript meets PLOS ONE's style requirements, including those for file naming. The PLOS ONE style templates can be found at

[https://journals.plos.org/plosone/s/file?id=wjVg/PLOSOne_formatting_sample_main_body.pdf](https://journals.plos.org/plosone/s/file?id=wjVg/PLOSOne_formatting_sample_main_body.pdf) and

[https://journals.plos.org/plosone/s/file?id=ba62/PLOSOne_formatting_sample_title_authors_affiliations.pdf](https://journals.plos.org/plosone/s/file?id=ba62/PLOSOne_formatting_sample_title_authors_affiliations.pdf)

Reply: We accepted your suggestion, and download the PLOS ONE style templates from the website as you noted. We revised manuscript to meets PLOS ONE's style requirements.

2. Please provide additional details regarding participant consent. In the ethics statement in the Methods and online submission information, please ensure that you have specified what type you obtained (for instance, written or verbal, and if verbal, how it was documented and witnessed). If your study included minors, state whether you obtained consent from parents or guardians. If the need for consent was waived by the ethics committee, please include this information.

Reply: we provided additional details regarding participant consent in our revised manuscript and supporting information files (S1).

3. We note that you have stated that you will provide repository information for your data at acceptance. Should your manuscript be accepted for publication, we will hold it until you provide the relevant accession numbers or DOIs necessary to access your data. If you wish to make changes to your Data Availability statement, please describe these changes in your cover letter and we will update your Data Availability statement to reflect the information you provide.

Reply: we agreed your suggestion

4. Your ethics statement should only appear in the Methods section of your manuscript. If your ethics statement is written in any section besides the Methods, please delete it from any other section.

Reply: we agreed your suggestion and deleted ethics statement appeared in the other section

5. We note you have included a table to which you do not refer in the text of your manuscript. Please ensure that you refer to Table 5 in your text; if accepted, production will need this reference to link the reader to the Table.

Reply: we thank very much for your suggest on Table 5. We confirmed the text of the Table 5 in revised manuscript.

6. Please include captions for your Supporting Information files at the end of your manuscript, and update any in-text citations to match accordingly. Please see our Supporting Information guidelines for more information: <http://journals.plos.org/plosone/s/supporting-information.>

Reply: After careful saw Supporting Information guidelines, we added captions for our Supporting Information files at the end of manuscript.

7. During our internal evaluation of the manuscript, we found significant text overlap between your submission and the following previously published works, some of which you may be an author:

[https://journals.sagepub.com/doi/10.1177/0733464810374468](https://journals.sagepub.com/doi/10.1177/0733464810374468)

- [https://www.cambridge.org/core/journals/international-psychogeriatrics/article/abs/lay-perceptions-about-mental-health-where-is-age-and-where-is-alzheimers-disease/08DD8B5191E8B6897AB3C24DA4E26BF7](https://www.cambridge.org/core/journals/international-psychogeriatrics/article/abs/lay-perceptions-about-mental-health-where-is-age-and-where-is-alzheimers-disease/08DD8B5191E8B6897AB3C24DA4E26BF7)

- [https://amhsjournal.org/article.asp?aulast=Bose&epage=235&issn=2321-4848&issue=2&spage=230&volume=8&year=2020](https://amhsjournal.org/article.asp?aulast=Bose&epage=235&issn=2321-4848&issue=2&spage=230&volume=8&year=2020)

- [https://www.semanticscholar.org/paper/Systematic-Review-of-Depression%2C-Anxiety%2C-and-Other-Dyrbye-Thomas/32c0691e8abd154312d5555193d0ff9fe224e5c4?p2df](https://www.semanticscholar.org/paper/Systematic-Review-of-Depression,-Anxiety,-and-Other-Dyrbye-Thomas/32c0691e8abd154312d5555193d0ff9fe224e5c4?p2df)

- [https://www.who.int/news-room/fact-sheets/detail/depression](https://www.who.int/news-room/fact-sheets/detail/depression)

We would like to make you aware that copying extracts from previous publications, especially outside the methods section, word-for-word is unacceptable. In addition, the reproduction of text from published reports has implications for the copyright that may apply to the publications.

Please revise the manuscript to rephrase the duplicated text, cite your sources, and provide details as to how the current manuscript advances on previous work. Please note that further consideration is dependent on the submission of a manuscript that addresses these concerns about the overlap in text with published work.

Reply: We thank you very much for the comments and suggestions on our manuscript. We invied the Charlesworth Author services to polish the manuscript. After that, we checked the manuscript word-for-word to ensure the quality .

**Reply to reviewer1**

We thank you very much for the constructive comments and suggestions on our manuscript. We have corrected our manuscript according to the comments.

 1.From the summary to the discussion, there are typographical errors, due to lack of spaces. For example, see lines: 40, 45, 50, 105, 106, 124, 136, 151, 158, 177, 179, 180, 181, 192, 200, 218, 222, 243, 245, 246, 247, 249, 251, 255, 256, 257, 287, 288.

On line 182 it says "SDD”, and I guess it should say "SDS".

Line 183, Table 2, say “meidcal”

The citations, particularly noted in the discussion, do not correspond to the references. For example, 35 says Liselotte and is Mao, 37 says Lloyd and is Camp, 38 says Camp and is Clark, 39 says Clark and is Chen, …

Reply: We thank you very much for the comments and suggestions on our manuscript. We checked and corrected the manuscript word-for-word to ensure the quality. The errors noted above have been corrected, and showed in the manuscrip.

2.Abstract: The results in terms of students' knowledge about depression are not observed

Reply: we added text related the results in terms of students' knowledge about depression in abstract section, and description showed in the manuscript as follows: There were statistical differences between the with depression students and the no depression students in question: “Females has more probability”, “Depression can be adjusted by oneself,” “Associated with one`s character,” “Know cure method of depression,” “Know drug use of depression,” “Know depression influence for health,” and “Know prevention method of depression.”

3. Introduction

1) The objectives of the study are not clear; they seem to be only descriptive but in their results, they present data of associations that are not indicated as objectives.

2) It is missing that they indicate the hypotheses (and their foundation) of the main associations of the study. For example, the authors could point out how knowledge affects the type of coping, or how knowledge and coping are associated with the presence of depression (depending on the real objectives of the study).

Reply: We agree with this suggestion and have modified the throughout introduction section as appropriate. the text has been showed in manuscript.

4. Methods

Indicate the total number of students invited to participate and the response rate.

Reply: we agree and accept your suggestion, the description about “the total number of students invited to participate and the response rate” has been added as following “During the period of work, 2200 medical students were invited. By March 20, a total of 1931 questionnaires had been returned by respondents, and the response rate was 87.8%.”

It is not clear how the authors evaluated coping: by one or several questions? was/were open-ended or students could choose from multiple options?

Reply: we agree and accept your suggestion, the description about coping styles has been added as follows “The copying strategy was assessed by the question, “if you are affected by depression, which copying style would be chosen (multiple choice) ?” “No coping way,” “ask for help from friends,” “ search a book, ask for help from psychological consultation office in the college,” “see the doctor to hospital” had been listed choices.

In the statistical analysis, the authors indicate that they will present the percentage of people in each group (line 155), but it is not clear to which groups they refer.

Reply: we agree and accept your suggestion. After carefully consideration, we deleted the percentage of people in each group (line 155) .

5.Results

In point 3 of the results (line 186 to 192), the information that was already indicated in the method could be omitted.

Reply: we agree and accept your suggestion. We deleted the content you mentioned.

Tables

1) Check that the sentences begin with a capital letter.

Reply:we agree and accept your suggestion.We check our text and use capital letter in the right place.

2) The tables do not have a presentation order, that is, they present Table 2 first.

Reply:we agree and accept your suggestion. We relist all tables with correctly order.

3) In Table 2, the word "frequency" could be used instead of "number".

Reply:we agree and accept your suggestion. We use the word "frequency"in Table 2.

4) The tables do not specify which values are in parentheses. See Table 1 (columns 2 and 3), Tables 3 and 4.

Reply:we agree and accept your suggestion. After carefully consideration, we deleted the percentage of people in Table 1 (columns 2 and 3), Tables 3 and 4.

5.Discussion

Missing put a paragraph with the conclusions.

Reply:we agree and accept your suggestion. We add the conclusion paragraph in our manuscript.

6.Check references.

Reply:we agree and accept your suggestion. After carefully revision, we relist and check all references in our manuscript.

**Reply to reviewer2**

We thank you very much for the constructive comments and suggestions on our manuscript. We have corrected our manuscript according to your comments.

1.Introduction

-The intro overly focuses on presenting depression statistics and prevalence rates. However, this needs further development in order to effectively build a line of argument. For example, the authors could explore and expand on factors linked/related to the observed increased prevalence rates in college/university students as the population of interest for the current study.

Reply: We agree with this suggestion and have modified the throughout introduction section as appropriate. the text has been showed in manuscript.

-More is needed to contextualise the study in the geographical context within which the study was conducted. It needs to be clear what the state of knowledge is around depression within China, and in particular among Chinese university students.

Reply: We agree with this suggestion and have modified the throughout introduction section as appropriate. the text has been showed in manuscript.

-The literature on treatments for depression is presented in an entirely descriptive manner, and it is not directly relevant to the present study

Reply: we agree with this suggestion. We deleted the content about treatments for depression.

-The rationale for the present study is not clear. The introduction has not managed to make a case for why this particular study is needed, in this particular context, and within this particular population. It is not clear what the gap in knowledge is, that the present study aims to fill.

The current study aimed to ascertain medical students` depression situation and their levels of knowledge about depression, then subsequently explore the relationship between level of knowledge and coping style of the medical students on depression.

-Lines 98-100 need clarifying. It is not clear what the conflict being referred to here is. It is also not clear how the present study aims to resolve this conflict. The point about distinguishing depression in the population lacks clarity.

-Lines 100-103 need to be further expanded in order to help develop a rationale for the present study. In what way is gathering this information beneficial? The authors need to explicitly state how knowledge of the role of these factors would be beneficial in advancing the knowledge base further.

Reply:we agree with 4 suggestion you noted. After carefully revision, the rationale for the present study were represent as follows “ Therefore, we found a conflict between the high rate of depression and the low rate of treatment. The key point is to distinguish depression in the population. To date, limited attention has been directed at individuals` knowledge, beliefs, and ways of coping with depression. Exploration of such knowledge and coping style(s) for depression may reveal factors related to the conflict. ”

2.Methods

-A summary of demographic information for participants should be reported under a ‘Participants’ sub-heading (not ‘respondents’).

Reply:we agree and accept your suggestion. We use the word "Participants"in the part of summary of demographic information for participants.

-The knowledge and coping style measure (lines 123-130) needs to be described more clearly. Including a couple of sample items will be helpful. It is unclear whether ‘knowledge’ and coping style are two separate subscales. As this is a newly developed measure, internal consistency and reliability/validity analyses should be reported. The scoring criteria is not described in an accessible manner.

Reply: we agree and accept your suggestion, the description about coping styles has been added as following “The copying strategy was assessed by the question, “if you are affected by depression, which copying style would be chosen (multiple choice) ?” “No coping way,” “ask for help from friends,” “ search a book, ask for help from psychological consultation office in the college,” “see the doctor to hospital” had been listed choices.

The part “knowledge about depression” was only a questionnaire designed by ourself,not a scale, so,we did not assess internal consistency and reliability/validity

3.Results

-Lines 180-182- reports factors ‘associated’ with higher SDS score. This needs revising for clarity, as no tests of association were conducted

Reply:we agree and accept your suggestion. The description you mentioned has been corrected as follows “ The medical students with the characteristic of sophomore (p=0.003), female (p=0.006), and poor family relationship (p<0.001) have higher SDD scores”

-Lines 186-200- the description of the scoring for the depression knowledge measure should be clarified and placed in the ‘Method’. It is also not clear why analyses were conducted for individual items of the measure.

Reply: we agree and accept your suggestion.We deleted the content you mentioned. The aims to analysis individual items was to found which knowledges were short for medical students.

-Lines 217-229- the results reported here are inaccessible. It is not clear what the figures are referring to.

-Lines 224- 237- logistic regression results are difficult to decipher.

-Table 5 has not been referenced within the text.

Reply: we agree and accept your suggestion.We show the results you mentioned by Table5. We corrected the text of logistic regression results in our manuscript as follows “Coping styles for depression were dichotomous. The ways of “ask for help from friends,” “search a book,” “ask for help from psychological consultation office in the college,” and “see the doctor to hospital” were defined as “coping with,” while “no coping way” was defined as “no to coping with.” Then, coping styles were used as dependent variables, and gender, age, specialty, grade, place of residence, one-child family, with good family relationship, and depression knowledge score were classed as independent variables. Multivariate logistic regression models were conducted in medical students with depression. Female (OR=1.470, 95%CI: 1.078, 2.005), residence in rural area (OR=1.496, 95%CI: 1.038, 2.156), poor family relationship (OR=2.428, 95%CI: 1.790, 3.293), and lower cognitive level of depression (OR=1.920, 95%CI: 1.426, 3.226) showed significantly relationship with “no to coping with” (Table 5).”

Discussion

- Lines242-261- provide comparisons in prevalence rates between present and prior studies. It is not clear whether the prior studies were conducted in medical students specifically. And if this is the case, lines 251-261 are redundant as the reasons for high prevalence rates presented here are applicable to all medical students. **The authors need to specifically explore reasons for differences in prevalence that are unique to Chinese medical students as compared to medical students from other countries.**

Reply: Studies mentioned in lines 242-261 were conducted in medical students specifically, so, the reasons were similar to other countries.

-Lines 294-296 & Lines 303-306 need clarifying

Reply: we agree and accept your suggestions. We modify the parts you mentioned in manuscript.

-Limitations need to be much more substantive. For example, it is not clear how utilising a single measure of depression was a limitation. It is also not clear what the limitations of self report alluded to are.

Reply: we agree and accept your suggestions. We modify the part of limitations according your suggestions.

Once again, thank you very much for your constructive comments and suggestions which would help us both in English and in depth to improve the quality of the paper.
