# Supplementary material for: Knowledge and coping style about depression in medical students: A cross-sectional study in China
Source: PLoS One. 2023 Oct 27;18(10):e0293511. doi: 10.1371/journal.pone.0293511 (PMC10610079; doi:10.1371/journal.pone.0293511)
Supplement: Attachment — Submitted filename: Response to Reviewers.docx [file pone.0293511.s005.docx]

**Our responds to the** **editor’s and the reviewer’s comments：**

**Reply to editor**

We thank you very much for the constructive comments and suggestions on our manuscript. We have corrected our manuscript according to the comments.

1. Please provide version of the items in the Supporting Information translated into English to allow for editorial review (Column headers in the ‘Data’ file and the ‘Questionnaire’).

Reply: we translated the items in the Supporting Information into English and can find in the Supporting Information.We named the Supporting Information using PLOS ONE conventions.

2. PLOS ONE does not copyedit accepted manuscripts, so the language in submitted articles must be clear, correct, and unambiguous. We recommend that authors seek independent editorial help before submitting a revision. These services can be found on the web using search terms like “scientific editing service” or “manuscript editing service.”

Reply: We thank you very much for the comments and suggestions on our manuscript. We invited the friend coming from University Of Glasgow to polish the manuscript. After that, we checked the manuscript word-for-word to ensure the quality .

3. Please ensure that the study is reported according to the STROBE guideline, and include the completed STROBE checklist as Supporting Information.  Please add the following statement, or similar, to the Methods: "This study is reported as per the Strengthening the Reporting of Observational Studies in Epidemiology (STROBE) guideline (S1 Checklist)."

The STROBE guideline can be found here: [http://www.equator-network.org/reporting-guidelines/strobe/](http://www.equator-network.org/reporting-guidelines/strobe/)

When completing the checklist, please use section and paragraph numbers, rather than page numbers.

Reply: we reported the completed STROBE checklist as Supporting Information and add the following statement to the methods: "This study is reported as per the Strengthening the Reporting of Observational Studies in Epidemiology (STROBE) guideline (S1 Checklist)."

4. Your Financial Disclosure from the submission form does not match the ‘Funding’ section at the end of the manuscript. Please ensure both are correct.

Reply: we revised the Financial Disclosure at the end of the manuscript.

**Reply to reviewer1**

We thank you very much for the constructive comments and suggestions on our manuscript. We have corrected our manuscript according to the comments.

1.Table 1

I do not understand why the authors decided to eliminate the percentages, I hope my observation has not been misunderstood. In case of putting the percentages again, it would be advisable to put them without decimals.

It would be advisable to leave the values of the statistics (chi square and OR [but not the “p” values]) with two decimal places.

From my point of view, the standards give clarity to the table. Why remove them, did any reviewers suggest it?

Reply: We thank you very much for the comments and suggestions on our manuscript. We checked and corrected the Table 1. We added the percentages without decimals and left the values of the statistics with two decimal places in all the tables in our manuscript.

2.Table 3

Be more detailed in the title, i.e. point out "what is associated with what" and/or be more specific in the table footnote.

Put percentages without decimals. Take care that it is all the data that shows a frequency.

Present the values of the statistics (X2 and OR) only with two decimal places.

Reply:We checked and corrected the title and heading of Table3.

3.Also, a typographical error check is again recommended, as in "sofeware" (Line 148)

Reply: The errors noted above have been corrected in the manuscript.

**Reply to reviewer2**

We thank you very much for the constructive comments and suggestions on our manuscript. We have corrected our manuscript according to your comments.

1.Abstract

• Line 43-48- the observed differences between groups (with and without depression need clarifying).

Reply: We checked and corrected the line 43-48 as following “ There were statistical differences between with depression students and no depression students on the rate of the correct answer in the following questions:” in the abstract part of the manuscript.

• Lines 45-53- it is not clear what the dependent variable being predicted here is

Reply:The dependent variable being predicted in Lines 45-49 was “on the rate of the correct answer”.

The dependent variable being predicted in Lines 51-53 was “the coping way”.

• The conclusion is not clear, and none of the statements made here are accessible. For example, does the reference to ‘broadcasting mental health’ infer the importance of sharing whether one has mental health problems? It is not clear.

Reply:We thank you very much for the constructive comments and suggestions on our manuscript. We have corrected conclusion as following “It is necessary to focus on mental health of medical students, especially in female, residence in rural area, poor family relationships, and lower cognitive level of depression knowledge. Medical students were insufficient on depression knowledge and coping styles, efforts that train students know risk of impaired mental health could also improve diagnosis and treatment.”

2. Introduction

• Typos- line 70 page 3- should be ‘depression’ not depressing; line 86 page 4 ‘made no treated history’- should this be ‘had no history of treatment’?

Reply: We agree with this suggestion and modified the two sections in our manuscript.

• The introduction is very brief, and lacking in analysis of the presented information. For example, page 3 line 78 could comment on and explore reasons for the significant differences in prevalence rates (6-66.5%).

Reply: We added some comments in the part of introduction.

3.Method

• Page 4 line 104- should read ‘the questionnaire survey’ or simply ‘the survey’

Reply: We agree with this suggestion. We corrected the express with ‘the questionnaire survey’.

• Arrangements for informed consent are slightly concerning. It is not clear how consent was informed based only on a ‘statement’ particularly given the sensitive nature of the study. Did participants explicitly consent (e.g. by ticking a box) or was consent inferred by progressing with questionnaire? How much information about the study did they receive prior to completing the questionnaires?

Reply: We thank you very much for the constructive comments and suggestions on our manuscript. Arrangements for informed consent as following: “The questionnaire included a statement about informed consent at the beginning. The participant was asked to answer the question “are you still willing to participate in our survey after read and understand our research intentions?” Only those who chose "yes" were considered consent to write out the questionnaire.”

• Page 6 line 148 typo- should be ‘software’

Reply: The errors noted above have been corrected in the manuscript.

4.Results

• Table 3 needs clarifying. For example, the % of correct answers column- percentages are in brackets but not clear what the number appearing before this is? Is this number of correct answers? Does the ‘with depression’ and ‘no depression’ colums show the number in each group endorsing a correct answer? This is not clear. Finally was the chi square comparing absolute numbers in each group (depression v no depression) endorsing the correct answer or the proportions? This needs explaining in the main body. It also needs to be clear what the significant results show.

Reply: We checked and corrected the title with “ Number of correct answer about the knowledge of depression in medical students”, which indicated “with depression’ and ‘no depression’ colums show the number in each group endorsing a correct answer” , accordingly, the heading of table3 also been corrected.

• Logistic regression results- the concept of ‘no to coping with’ which is the outcome variable needs clarifying as it is not accessible- i.e. not clear what it means.

Reply: Only the medical students with depression conducted the Logistic regression analysis, coping styles for depression was the dependent variable.

• Multiple comparisons increase risk of family-wise error rate. Thus Bonferroni correction is needed for all analyses.

Reply:We agree with this suggestion. Bonferroni correction was used to all analysis in our manuscript.

5. Discussion

• Page 13 line 224-225 is directly contradicting lines 220-222.

Reply: :We agree with your suggestion. The content in lines 220-222 was deleted.

• Lines 225-227- not clear here whether the comparisons being discussed are of other medical students.

Reply: In Lines 225-227, the comparisons being discussed are of other medical students in related many studies. More details can find in our manuscript.

• Line 232- do you mean ‘wellness’

Reply: The errors noted above have been corrected.

• Linbe 268- do you mean ‘will be qualified’?

Reply: The errors noted above have been corrected with “will be able to”.

• Line 270- do you mean ‘could affect’

Reply: The errors noted above have been corrected with “could affect”.

• Line 271-272 (and throughout the paper)- you refer to ‘no coping way’- do you mean ‘no coping mechanisms’? or ‘no ways of coping’?

Reply:We agree with your suggestion. We used the expression “ no ways of coping” in full manuscript.

• Iine 272-273- it is not clear how your results compare to Thompson as there is a lack of clarity in regards to your conceptualisation of ‘no coping way’.

#### Reply: We agree with your suggestion.We used the expression “ no ways of coping”, meaning that no any ways to coping with depression or ignore in our manuscript. Our results are similar to Thompson`s.

• Lines 273-281- need revising for grammar

Reply:We agree with your suggestion. We revised the part carefully. More details can be find in our manuscript.

• Lines 292-296- not substantive. Multiple tools can still capture depression adequately, and there is never an expectation for a study to use all available tools

Reply:We agree with your suggestion. We deleted the content in the part of limitation.

• Lines 296-298- need revising for meaning

Reply: The content in lines 296-298 “among the variety of tools available to assess depression, this study used only SDS as a tool to assess symptoms of depression. The SDS scale is considered a good discriminat tool between depressed and non-depressed individuals, only can to compare with studies used the scale, can not compare with studies used other tools.” was deleted according to the full text .

Once again, thank you very much for your constructive comments and suggestions which would help us both in English and in depth to improve the quality of the paper.
