# Supplementary material for: Knowledge and coping style about depression in medical students: A cross-sectional study in China
Source: PLoS One. 2023 Oct 27;18(10):e0293511. doi: 10.1371/journal.pone.0293511 (PMC10610079; doi:10.1371/journal.pone.0293511)
Supplement: Attachment — Submitted filename: Response to Reviewers.doc [file pone.0293511.s006.doc]

**Our responds to the** **editor’s and the reviewer’s comments：**

**Reply to editor**

We thank you very much for the constructive comments and suggestions on our manuscript. We have corrected our manuscript according to the comments.

Please review your reference list to ensure that it is complete and correct. If you have cited papers that have been retracted, please include the rationale for doing so in the manuscript text, or remove these references and replace them with relevant current references. Any changes to the reference list should be mentioned in the rebuttal letter that accompanies your revised manuscript. If you need to cite a retracted article, indicate the articles retracted status in the References list and also include a citation and full reference for the retraction notice.

Reply: We review carefully our reference list, it is complete and correct, and no retracted reference were cited.

**Reply to reviewer1**

Reviewer #1: All comments have been addressed. I note some style errors that will surely be pointed out when editing the manuscript.

Reply: We thank you very much for the comments and suggestions on our manuscript. We corrected as far as possible in style errors and spelling mistake in our manuscript.

**Reply to reviewer2**

1. I would suggest that the author hires someone to correct the English language. It turns out that the previous version was much better than the update one when it comes to grammar and language editing.

Reply: We thank you very much for the comments and suggestions on our manuscript. We invide the English specialized staff to correct our manuscript .

2.On the titles of the summary tables, it is not clear what the Odds Ratio (OR) is about. Specifically, what is the outcome variable, and what is the model predicting? I suggest clearly distinguishing between crude OR and adjusted OR.

Reply: We thank you very much for the comments and suggestions on our manuscript. We added the note at the bottom of the table as follows “ it means that the size of risk on a characteristic between the medical students with depression and no depression.”

3.The selected sample may not represent the entire student population. Thus, the result may not be generalizable.

Reply: We thank you very much for the comments and suggestions on our manuscript. We highlighted the piont in the part of limitation as follows “ The medical students in our study were not be selected randomly, so, they didn't represent the entire student population in China, the result may have effect on generalization ”.

4. It would be nice to show the interval reliability measures of the responses.

Reply: We thank you very much for the comments and suggestions on our manuscript. We calculated the Cronbach's α for interval reliability in our manuscript.

5.Some formatting is needed for representing the OR with CI and similar places throughout the paper. For example, OR with confidence intervals are written as 1.027(0.744,1.418) There should be a space after 1.027 and after the comma, to look like the following: 1.027 (0.744, 1.418)

Reply: We thank you very much for the comments and suggestions on our manuscript. We corrected as far as possible in style errors and spelling mistake in our manuscript.

Once again, thank you very much for your constructive comments and suggestions which would help us both in English and in depth to improve the quality of the paper.
