# Supplementary material for: Knowledge and coping style about depression in medical students: A cross-sectional study in China
Source: PLoS One. 2023 Oct 27;18(10):e0293511. doi: 10.1371/journal.pone.0293511 (PMC10610079; doi:10.1371/journal.pone.0293511)
Supplement: Attachment — Submitted filename: Response to Reviewers.doc [file pone.0293511.s007.doc]

**Our responds to the reviewer’s comments：**

**Reply to reviewer4**

We thank you very much for the constructive comments and suggestions on our manuscript. We have corrected our manuscript according to the comments.

1. The language used lacks academic rigor and coherence, which affects the clarity and precision of the text.

Reply:We thank you very much for the comments and suggestions on our manuscript. We invide the English specialized staff to correct our manuscript .

2. There are errors in the expression of some statistical symbols used in the study, such as the chi-square value. It is recommended to carefully review and correct these errors.

Reply: We thank you very much for the comments and suggestions on our manuscript. We corrected as far as possible in some statistical symbols used in our manuscript.

3. The selection of keywords and the phrasing of the title may benefit from further optimization to accurately and effectively communicate the study's focus and contribution.

Reply: We thank you very much for the comments and suggestions on our manuscript. We corrected some keywords in our manuscript.

4. The current research lacks substantial depth in both its content and analysis.

Reply: We thank you very much for the comments and suggestions on our manuscript.We added content in the part of discussion.

Once again, thank you very much for your constructive comments and suggestions which would help us both in English and in depth to improve the quality of the paper.
