# Supplementary material for: Knowledge and coping style about depression in medical students: A cross-sectional study in China
Source: PLoS One. 2023 Oct 27;18(10):e0293511. doi: 10.1371/journal.pone.0293511 (PMC10610079; doi:10.1371/journal.pone.0293511)
Supplement: Attachment — Submitted filename: Response to Reviewers.doc [file pone.0293511.s008.doc]

**Our responds to the reviewer`s comments：**

**Reply to reviewer5**

We thank you very much for the constructive comments and suggestions on our manuscript. We have corrected our manuscript according to your comments.

1. Please clarify the number of respondents excluded and the reasons for exclusion to provide transparency in sample selection.

Reply:We thank you very much for the comments and suggestions on our manuscript. We added the description about the number of respondents excluded and the reasons in 119-123 line as following: During the period of work, 2200 medical students were invited. Each invited medical students could complete the questionnaire only once. By March 20, a total of 1931 questionnaires had been returned by respondents, and the response rate was 87.8%. 269 invited no returned the questionnaire, the reasons may be disagree or miss the time. Questionnaires that had completed less than 100 seconds were considered unusable, 1909 questionnaires were usable, and 22 were removed from the analysis.

2. Provide more details about the demographic characteristics (e.g., age range, gender distribution) to enhance the understanding of the study population.

Reply:We thank you very much for the comments and suggestions on our manuscript. “age range, gender distribution” have been showed in 176-177 line in our manuscript.

3. Include mean and standard deviation values for continuous variables (e.g., age, SDS score) in addition to frequencies.

Reply:We thank you very much for the comments and suggestions on our manuscript. “mean and standard deviation of age ” have been showed in 176 line. “mean and standard deviation of SDS score” have been showed in table 2 in our manuscript.

4. Provide a more comprehensive discussion of the findings, including comparisons with previous studies and an analysis of the implications for medical education and student support services. Discuss the potential reasons behind the high prevalence of depression among medical students in China, considering factors such as academic pressure, long study duration, and clinical practice stress.

Reply:We thank you very much for the comments and suggestions on our manuscript. A more comprehensive discussion including you mentioned above have been showed in 249-256 line and 312-314 line in our manuscript.

5. Address the limitations of the study more explicitly, including the potential biases associated with self-reporting surveys and the need for further research in different regions of China.

Reply:We thank you very much for the comments and suggestions on our manuscript. A more comprehensive discussion including you mentioned above have been showed in 318-324 in our manuscript.

Once again, thank you very much for your constructive comments and suggestions which would help us both in English and in depth to improve the quality of the paper.
